# Proprioceptive loss and the perception, control and learning of arm movements in humans: evidence from sensory neuronopathy

**DOI:** 10.1007/s00221-018-5289-0

**Published:** 2018-05-19

**Authors:** R. Chris Miall, Nick M. Kitchen, Se-Ho Nam, Hannah Lefumat, Alix G. Renault, Kristin Ørstavik, Jonathan D. Cole, Fabrice R. Sarlegna

**Affiliations:** 1 School of Psychology, University of Birmingham, Birmingham, B15 2TT UK; 2 Department of Neuroscience, University of Pennsylvania, Philadelphia, USA; 3 Aix Marseille Univ, CNRS, ISM, Marseille, France; 4 Department of Neurology, Oslo University Hospital, Oslo, Norway; 5 Centre of Postgraduate Research and Education, Bournemouth University, Bournemouth, UK

**Keywords:** Human movement, Proprioception, Deafferentation, Sensorimotor, Adaptation, Neuronopathy, Force-field adaptation, Limb dynamics, Vision

## Abstract

It is uncertain how vision and proprioception contribute to adaptation of voluntary arm movements. In normal participants, adaptation to imposed forces is possible with or without vision, suggesting that proprioception is sufficient; in participants with proprioceptive loss (PL), adaptation is possible with visual feedback, suggesting that proprioception is unnecessary. In experiment 1 adaptation to, and retention of, perturbing forces were evaluated in three chronically deafferented participants. They made rapid reaching movements to move a cursor toward a visual target, and a planar robot arm applied orthogonal velocity-dependent forces. Trial-by-trial error correction was observed in all participants. Such adaptation has been characterized with a dual-rate model: a fast process that learns quickly, but retains poorly and a slow process that learns slowly and retains well. Experiment 2 showed that the PL participants had large individual differences in learning and retention rates compared to normal controls. Experiment 3 tested participants’ perception of applied forces. With visual feedback, the PL participants could report the perturbation’s direction as well as controls; without visual feedback, thresholds were elevated. Experiment 4 showed, in healthy participants, that force direction could be estimated from head motion, at levels close to the no-vision threshold for the PL participants. Our results show that proprioceptive loss influences perception, motor control and adaptation but that proprioception from the moving limb is not essential for adaptation to, or detection of, force fields. The differences in learning and retention seen between the three deafferented participants suggest that they achieve these tasks in idiosyncratic ways after proprioceptive loss, possibly integrating visual and vestibular information with individual cognitive strategies.

## Introduction

Flexible and adaptive motor control requires optimal integration of all available sensory inputs (Kording and Wolpert 2004; Scott 2004; Bays and Wolpert 2006). Still to be resolved is the extent to which adaptation and control are compromised by loss of vision and proprioception. Adaptation to new limb dynamics has been shown to be similar with or without vision, in normal participants, suggesting that proprioception is sufficient (Scheidt et al. 2005; Franklin et al. 2007; Lefumat et al. 2015; see also DiZio and Lackner 2000). Experiments after recent pathological loss of proprioception have not been possible; such participants are not readily available and their motor control appears initially so compromised as to make experiments difficult. However, adaptation to imposed changes in visual feedback is possible in the chronic absence of proprioception (Bard et al. 1995; Ingram et al. 2000; Bernier et al. 2006; see also surgically deafferented, non-human primate studies: Bossom and Ommaya 1968). Successful visuomotor adaptation is thought to reflect an updated internal model of the relationship between visual goals and motor commands. In fact, the few participants with chronic proprioceptive loss (PL) assessed in these circumstances are remarkably adept, when allowed vision of their actions (Blouin et al. 1993; Fleury et al. 1995; Ghez et al. 1995; Lefumat et al. 2016), and adapt their movements as much as normal subjects, albeit with cognitive effort (Ingram et al. 2000) and without efficient inter-joint coordination (Sainburg et al. 1995). They can also adapt to novel tools which extend arm length and thus alter the normal kinematics (Cardinali et al. 2016).

However, adaptation to new forces acting on the limb, while possible with visual feedback (Sarlegna et al. 2010; Yousif et al. 2015; Lefumat et al. 2016), would seem a more challenging task for an individual with PL (Sainburg et al. 1995; Pipereit et al. 2006; Bock and Thomas 2011). In the absence of limb proprioception, a perturbation such as an unexpected force is most likely perceived via visual feedback, as the distorted trajectory and/or the mis-reached goal would provide a visual error signal. For position-dependent forces, such as springs, the forces are present and large at the end of the movement, and so readily apparent at movement end. However, velocity-dependent forces are only present during motion. It is, therefore, remarkable that PL participants can not only adapt to these forces (Sarlegna et al. 2010; Lefumat et al. 2016), but also seem to do so selectively (Yousif et al. 2015), such that their responses to either position-dependent or velocity-dependent forces are distinct and appropriate to the perturbation profile.

What features of the visual feedback used in dynamic adaptation is not yet known, but the adaptation processes could be driven by comparison between the planned trajectory and the observed movement: indeed, as the forces are often introduced abruptly, they lead in the first few trials to substantial trajectory errors which can be easily observed by the participant (Brashers-Krug et al. 1996; Sarlegna et al. 2010; Lefumat et al. 2016). Lago-Rodriguez and Miall (2016) recently showed that normal subjects actively de-adapt learned responses based on “errorless” visual feedback, supporting the idea that de-adaptation, and most likely adaptation, is driven by the difference between an internally predicted trajectory and visual feedback. Melendez-Calderon et al. (2011) have shown complementary adaptation to virtual curl-field forces during movements made without any proprioceptive error, driven by a virtual visual error. Thus, both visual and proprioceptive prediction errors can drive adaptation.

Several recent studies have reported significant correlations between individuals’ kinematics and their degree of adaptation. For example, Wu and colleagues (2014) showed that greater movement variability during baseline predicted better subsequent learning, in both visuo-motor and force-field adaptation tasks. They also showed that training reshaped the profile of this variability across different tasks, implying its active regulation (see also Pekny et al. 2015). Wong and Shelhamer (2014) reported similar relationships between baseline errors and adaptation of saccadic eye movements, while Lefumat et al. (2015) showed that variability of arm movement during the adaptation phase was correlated with the amount of inter-manual transfer of force-field adaptation, a finding that also held up for two participants with PL (Lefumat et al. 2016).

One argument is that the individual differences in kinematics expose the properties of the learning process. For example, high variability during baseline might reflect an exploratory strategy and might predict high variability during early adaptation, which would complement error-driven learning to achieve strong adaptation (Wu et al. 2014). Likewise, considering the proposal of fast and slow processes underlying sensorimotor adaptation (Smith et al. 2006; Trewartha et al. 2014; Huberdeau et al. 2015), higher variability might drive greater change in the fast process, while the slow process would be relatively immune to trial-by-trial variation (Baddeley et al. 2003; Huang and Shadmehr 2009). Other possibilities are that individuals plan idiosyncratic trajectories, or differ in their ability to detect a mismatch between planned and executed trajectories, thus leading to different levels of adaptation (Kanai and Rees 2011; Seidler et al. 2015; Raket et al. 2016; Christou et al. 2016).

We aimed here to address several of these issues by testing sensorimotor adaptation to dynamic force fields in three chronically deafferented participants, two of whom have been tested previously in different protocols. The three PL participants were first tested in adaptation to a single curl-field, and then tested with the double-force-field protocol used to assess dual-rate learning (Smith et al. 2006). We measured during adaptation both their directional reaching errors and their compensatory forces. We also modeled their performance, to test where differences may lie compared to normal participants, and we tested for the expected predictive relationships between movement kinematics and adaptation. Finally, we assessed their ability to detect the force fields with and without visual feedback, to determine whether other, non-visual, cues may be available to assist their performance in these tasks. Our hypothesis was that PL participants can adapt to a velocity-sensitive force field with the help of visual feedback (Sarlegna et al. 2010; Yousif et al. 2015). Since they tend to produce more variable movements than controls, and rely heavily on cognitive strategies, we expected to find a greater contribution of the fast learning process compared to controls (Smith et al. 2006; Wu et al. 2014; Taylor et al. 2014). On the assumption that they would be more reliant on strategic, cognitive mechanisms than normal controls, and less influenced by slow, implicit learning, we predicted no spontaneous recovery of slow memory after the double-adaptation exposure. We also hypothesized that detection of force-field direction would be possible for PL participants with visual feedback but would be greatly impaired or impossible when deprived of this crucial information.

## Methods

We first describe the common features across the three experiments, while specifics for each experiment are detailed below.

### Participants

#### Deafferented patients

Three participants who suffer from a chronic, stable sensory neuronopathy participated in these experiments. All three have a specific, massive loss of large, myelinated sensory fibres. IW (male, 61 years old at time of participation, 100% left-handed according to the 10-item version of the Edinburgh inventory, Oldfield 1971) has no tactile, muscle or joint proprioceptive sensation from below the collar (C3) following a selective peripheral neuronopathy brought about by a viral infection at age 19 years. GL (female, 66 years old at time of participation, right-handed with a lateral quotient of 77%; Lefumat et al. 2016) suffered a similar neuronopathy except the loss of sensation is from the mouth down (trigeminal V3, Forget and Lamarre 1987; Cole and Paillard 1995). IW has apparently normal afferent sensations from neck muscles whilst GL has no afferent information from neck muscles or lower face. Efferent motor and afferent pain and temperature fibres remain intact, as are vestibular sensations of head position in space. Detailed case histories for IW and GL have previously been described (Cole and Sedgwick 1992; Cole and Paillard 1995; Forget and Lamarre 1995). GL never regained the ability to walk unaided, and she abandoned it because of the very high demands it imposed, finding that using a wheelchair gave her much more freedom of activity and an ability to carry out a near normal family life; GL remains ataxic. IW has largely diminished ataxia and moves cautiously and accurately. IW did walk unaided for many years (Lajoie et al. 1996) but now uses a wheelchair to move more than a few metres. It should be emphasized that over more than two decades both IW and GL have participated in many reaching studies and are probably performing at a much higher level than those newly with proprioceptive loss could. A third participant, WL (female, 46 years old at time of participation, left-handed) was also recruited. She has not been a participant in sensory or motor experiments before and is less extensively neurophysiologically characterized than IW or GL. WL was diagnosed with acute polyradiculitis at the age of 31. She was paralyzed and with total loss of deep sensation and touch. She regained muscle strength gradually over 1 month. However, as a sequela she has no deep sensibility from the third branch of the trigeminal nerve bilaterally and below and can only perceive some degree of light touch when tested with moving objects (e.g., Somedic brush). This kind of touch is also to some degree perceived as unpleasant, perhaps reflecting central sensitization. Electrophysiological testing showed initially some improvement of sensory responses, but when this was last retested with nerve conduction velocity testing, 6 years prior to these experiments, there were no clear sensory responses in the upper or lower extremities. However, quantitative sensory testing reveals normal thresholds for temperature, indicating a sparing of small C and A-delta nerve fibres. WL is more ataxic and uncertain in all her movements than IW or GL. She is wheelchair-bound and never regained the ability to walk after she fell ill 18 years ago, despite normal motor strength.

#### Control participants

Five separate groups of control participants were recruited. Demographic details are provided in the relevant sections below.

#### Ethics

The University of Birmingham STEM ethics committee approved all experiments. All participants were provided with appropriate information about the task prior to the experiment, and gave their written consent, according to the Declaration of Helsinki.

## Experimental setup

**Fig. 1:**
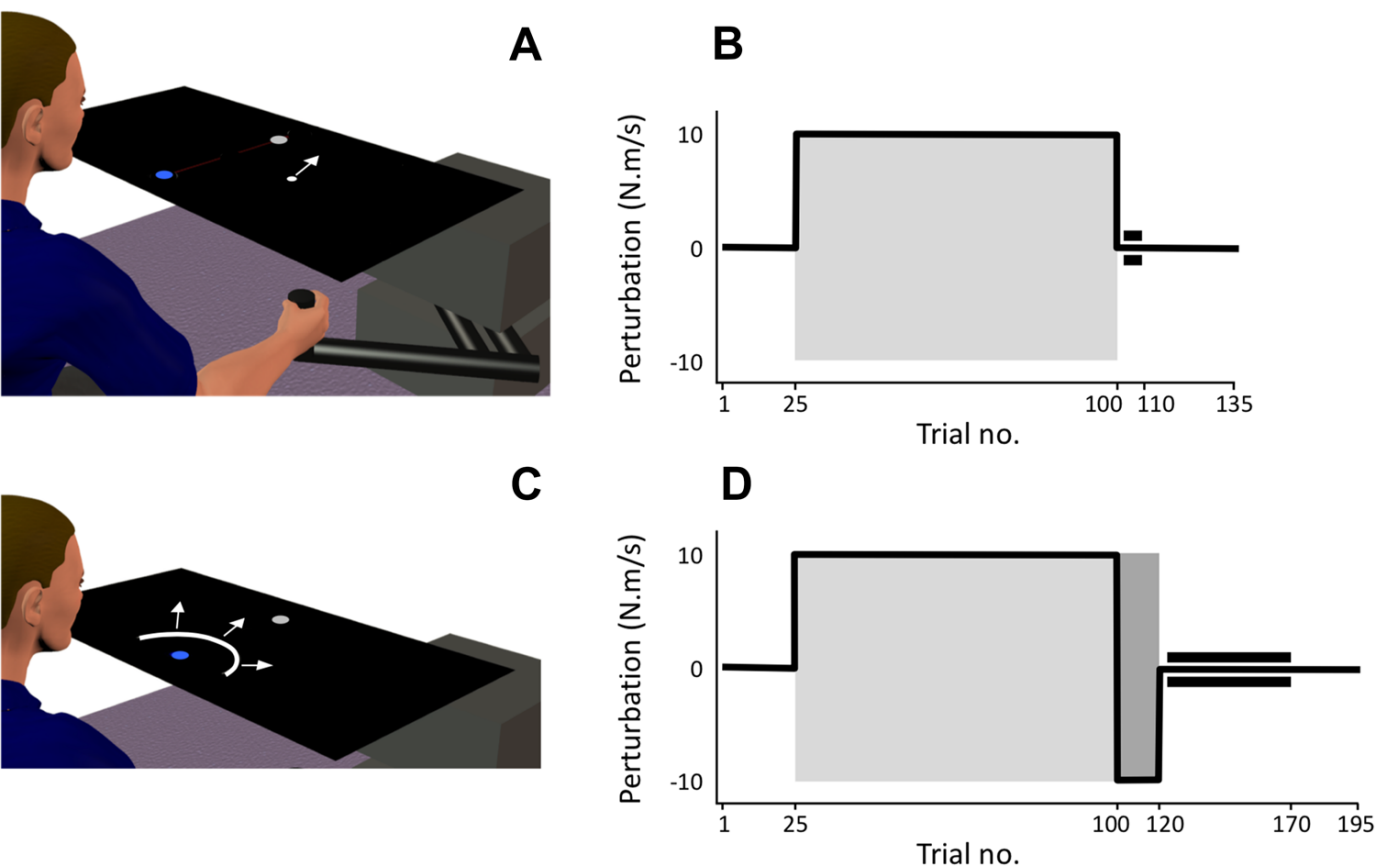
**a** Schematic representation of the setup. Participants viewed a display reflected in a horizontal mirror so that the image appeared in the plane of the hand movement. They held the robot handle with their preferred hand and reached from a nearby start circle (blue) to a distal target (grey). The white cursor (represented with an arrow) moved as it accurately displayed the hand position. **b** Timeline for experiment (1) After a short practice block (not shown), participants performed a baseline phase (25 trials), adaptation (75 trials), a retention phase (10 trials), and a final wash-out phase (25 trials); 1 in 5 trials in each phase was a channel trial, except for the retention phase which was all channel trials (as indicated by the tram lines). The grey box indicates the phase with applied curl-field forces. **c** In channel trials, the robot constrained movement to a straight channel, while visual feedback of distance only was provided with an expanding arc. **d** Timeline for experiment (2) After initial adaptation, there was a short reverse-force phase (20 trials), followed by 50 channel trials and a final wash-out phase

Participants were seated comfortably in front of a 2D-planar robotic manipulandum (vBOT; Howard et al. 2009), which they grasped with their dominant hand (Fig. 1). The handle could freely rotate around its vertical axis and the vBOT has been designed to have high stiffness and low mass, friction and viscosity at the end point (Howard et al. 2009): static friction is 0.15 N, the effective end-point mass varies with direction between 0.4 and 0.7 kg, and viscosity is approximately of 3.5–5.7 N/m/s. A padded chin rest (GL), forehead rest (IW and all controls), or wheelchair head support (WL) was used to stabilise the head. Participants were instructed to look down onto a mirrored surface, where they saw the reflected image of a flat-screen monitor display above (30-inch Apple Cinema HD; 70 Hz refresh rate). The virtual image was in the same plane and calibrated with the top surface of the robot handle (i.e., at a level about 2 cm above the participant’s thumb). The combined workstation comprising robot, mirror, monitor and frame could be raised vertically, and all three PL participants performed the experiment in their own wheelchairs, whose wheels were locked.

The display mirror blocked any direct vision of the hand or arm, although participants could initially grasp the handle with vision, by withdrawing their head from the chin or head rest. Visual feedback of the robot handle position could be controlled by the presence or absence of a white 1 cm diameter cursor whose position was updated in real time. Trial-by-trial robot handle position and translational force data were recorded at 1 kHz and stored for offline analysis.

### Common aspects of the motor tasks

Participants were required to move the vBOT handle position to bring the cursor to a 1 cm radius start position located 10 cm into the 40 × 64 cm workspace (approximately 30 cm from the participant’s torso) at which point the blue start circle turned green. When this position had been maintained for a 0.5–1.0 s random wait time, a 1 cm radius grey target appeared 20 cm directly ahead and cued the participant to make a rapid reaching movement towards it. The force-controlled robot was programmed, across different experiments and trials, to allow free movement to the target, or to provide perturbing velocity-sensitive curl-field forces that deviated the handle orthogonal to the reaching direction (Shadmehr and Mussa-Ivaldi 1994), or to provide stiff lateral resistance, forming a virtual channel that allowed only movement along the channel and negligible movement in the orthogonal axis (Scheidt et al. 2000). It also provided a soft spring-force field that braked forward movement beyond the far edge of the target (5 N/cm) and then switched to guide the hand back to the start location following a minimum jerk trajectory with a spring force of 0.5 N/cm, a minimum duration of 2 s and maximum speed of 2 m/s. Across trials, 3 different types of visual feedback conditions were used: participants either had no visual feedback about hand position, saw a visual cursor which provided calibrated feedback of the hand position (Fig. 1a), or received visual feedback about movement distance in the form of a semi-circular arc that was centred on the start circle, indicating the extent but not the direction of their reaching movement (Fig. 1c; Lago-Rodriguez and Miall 2016). Because PL participants generally have deficits in control of movement extent in the absence of visual feedback (Gordon et al. 1995; Sarlegna et al. 2006), all participants had visual (arc) feedback about movement distance in channel trials (Fig. 1c). They also received arc feedback (in one of three 120-degree sectors) for the final 2 cm when returning to the start location. All participants were advised that the vBOT handle would perturb their reaching movements, on some trials, or that the visual feedback would change to an arc, although they were not informed about when these changes would happen. They were given a short practice session of 25 trials that included 5 force trials and 5 channel trials.

## Experiments 1 and 2: adaptation to force fields

### Deafferented participants

All three participated, in separate 2-day testing sessions held over a period of 10 months. GL, IW and WL each completed two adaptation sessions over 2 days.

### Control participants

Seven controls (5 male, 62.7 ± 1.6 years, 1 left-handed) also participated in the experiment (“control group A”), with their handedness assessed using the 10-item Edinburgh Handedness Inventory (laterality quotient > 30/100 = right-handed; Oldfield 1971). IW (61 years) was well matched for age with the controls (*t* score = − 1.1), GL (66 years) less so (*t* score = 2.0) while WL (46 years) was younger than the controls (*t* score = − 10.4).

### Behavioural tasks

We first ran a “single-adaptation” test on the three PL participants (experiment 1). Participants were instructed to reach as fast and as accurately as possible toward the visual target. After each trial the robot guided the hand back into start position. In a practice session, they first made 25 reaching movements to the target: 20 trials in the null field (4 blocks of 5 trials; no active forces, alternating blocks with and without a visual cursor) and then 5 curl-field trials; every fifth trial was a channel trial. Immediately after this practice, they performed 135 trials, comprising 25 in the null field (baseline), 75 with a curl-field (adaptation), 10 with channel trials only (retention), and a final 25 trials as a washout with null field (Fig. 1b). During the null field, curl field and wash-out phases, 1 in 5 trials was pseudo-randomly selected as a channel trial (Fig. 1c). Control participants were not tested on this task, which was intended to verify whether PL participants could adapt to a curl field with a cursor that provided feedback about hand position alone.

The curl-field imposed a velocity-dependent force field:

1 $$\left[ {~\begin{array}{*{20}{c}} {{f_x}~} \\ {{f_y}} \end{array}} \right]={\text{FS}} \cdot \left[ {~\begin{array}{*{20}{c}} 0&{1~} \\ { - 1}&0 \end{array}} \right]\left[ {~\begin{array}{*{20}{c}} {{v_x}~} \\ {{v_y}} \end{array}} \right]$$

where *v*_*x*_ and *v*_*y*_ are the handle velocities in *x* and y dimensions, and *f*_*x*_, *f*_*y*_ the imposed forces. The field strength (FS) was ± 10 N.m/s for Experiment 1; the sign (the curl-field direction) varied as described below. For the right-handed GL, the curl field was anti-clockwise. For the left-handed IW and WL, it was clockwise.

PL and control participants then performed a “double-adaptation” task (Experiment 2) based on that reported by (Smith et al. 2006) with a 25-trial practice session, and then 25 null-field trials (baseline), 75 curl-field trials (adaption in the “primary” curl-field direction), 20 secondary curl-field trials with an opposite curl-field (reverse), 50 channel trials (retention) and a final set of 25 wash-out trials (Fig. 1d). The direction of the primary curl-field was opposite to that experienced in the single-adaptation sessions; the direction of the secondary curl-field was opposite to that of the primary curl field. For the left handers (IW, WL and 1 control), the primary curl-field was anti-clockwise; for the right handers (GL and 5 controls) it was clockwise. For the null field, primary adaptation and wash-out phases, 1 in 5 trials was a channel trial, pseudo-randomly sequenced. For the PL participants, the double-adaptation session (Experiment 2) was held on the day following the single-adaptation test (Experiment 1). Between these experiments they also performed sessions of Experiment 3 (see below), which had clockwise and anti-clockwise forces applied in pseudorandom order, ensuring effective washout of any residual adaptation. The control group performed their practice and double-adaptation sessions a few minutes apart within 1 day.

### Analysis

Performance in the null field, adaptation and wash-out trials (feedback trials) was quantified by the angle of the reaching movement at peak velocity, relative to the start location; as humans tend to perform rectilinear movements (Hogan and Flash 1987), perfect performance is assumed to have an angular deviation of zero. For the channel trials, in which deviation from the straight-line path to the target was impossible, performance was quantified by the force delivered against the channel wall at peak velocity, normalised by the peak velocity (Scheidt et al. 2000; Lago-Rodriguez and Miall 2016); perfect compensation for the applied curl-field would then reach 10 N.m/s, equal to the curl-field strength. For display and analysis, angular and force data from participants tested with opposite curl fields have been inverted for left handers. We used standard analyses of variances with repeated measures (RM-ANOVAs) to analyse results from healthy subjects. We also report *t* tests to compare each individual PL participant’s data values to the control group sample.

### Modelling

We used a state-space model to estimate the four parameters of a dual-rate learning model (Smith et al. 2006), from the compensatory channel trials in Experiment 2, fitted to individual subjects to estimate error-driven learning and forgetting rates. To separately model the angular deviation data from feedback trials, a fifth free parameter was necessary to scale the force-field perturbation, because the relationship between imposed forces and measured kinematic deviation is idiosyncratic across participants. Model parameters were estimated with the *fmincon* function in Matlab, constraining the slow process to have higher retention and lower learning rate than the fast learner, while all learning/retention rate parameters were bounded between 0 and 1. To avoid local minima, initial parameter estimates were varied over 10 runs by adding random noise with gradually increasing variance, while individual model fits were iterated 20 times.

### Results: Experiment 1

#### Practice

**Fig. 2:**
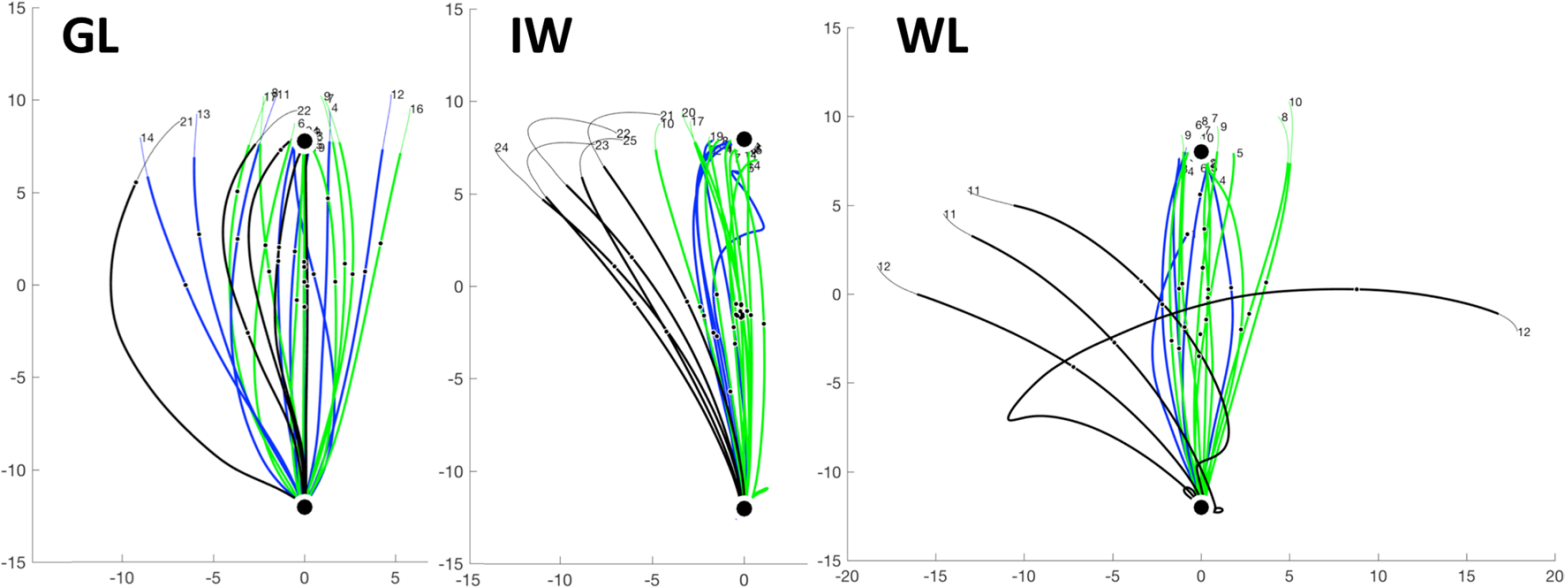
Experiment 1, movement trajectories for three PL participants during their first practice session. Each trace is the outward movement from the start location (at bottom of each panel) to the target at the top (large black dot); the axes are in centimetres, relative to the centre of the workspace. The full traces are shown in fine line, and the segment until the movement distance was exceeded is shown in thicker lines. The blue trials are null field with visual cursor feedback; green are null-field without visual feedback and black are trials with the curl field (in the opposite polarity to that used in the main test session). Channel trials were constrained to a straight-line trajectory and are not shown. Note that participant WL lost control of the handle on one curl-field trial. Other format details are as in Fig. 4

Figure 2 shows the initial practice trials for the three participants. Their movements in the null field were reasonably straight until they exceeded the target distance and were exposed to the braking forces of the robot; directional error and end position variance was higher than usual in controls (see Fig. 4). Strikingly, there was limited evidence of online correction for the curl-field (with visual feedback; black trials) and limited difference in null-field trials with and without visual cursor feedback (blue vs green).

#### Single adaptation

**Fig. 3:**
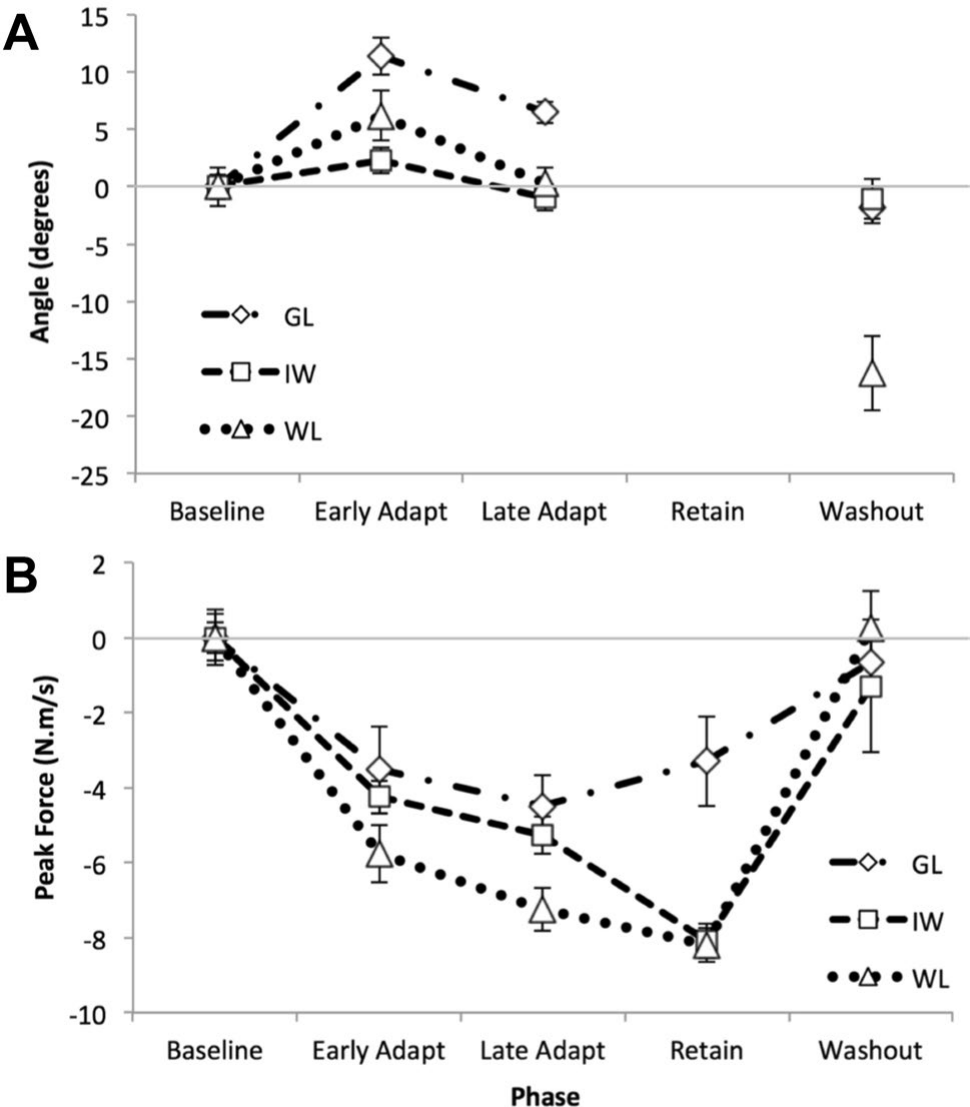
Experiment 1, deafferented participants only. **a** Mean deviation of the reaching trajectory, measured in visual feedback trials across the single-adaptation experiment. The adaptation phase (“Adapt”) was divided into two halves, early and late (37 trials each); there were no feedback trials in the retention (“Retain”) phase, between late adaptation and washout, which consisted only of channel trials. Each line represents the mean for one PL participant, ± 1 SEM. The individuals’ data have been normalised to their mean baseline performance. **b** Compensation for the curl-field force was measured in channel trials presented infrequently across the single-adaptation experiment (1:5 trials, pseudo-randomly, except for the retention phase which consisted only of channel trials)

**Fig. 4:**
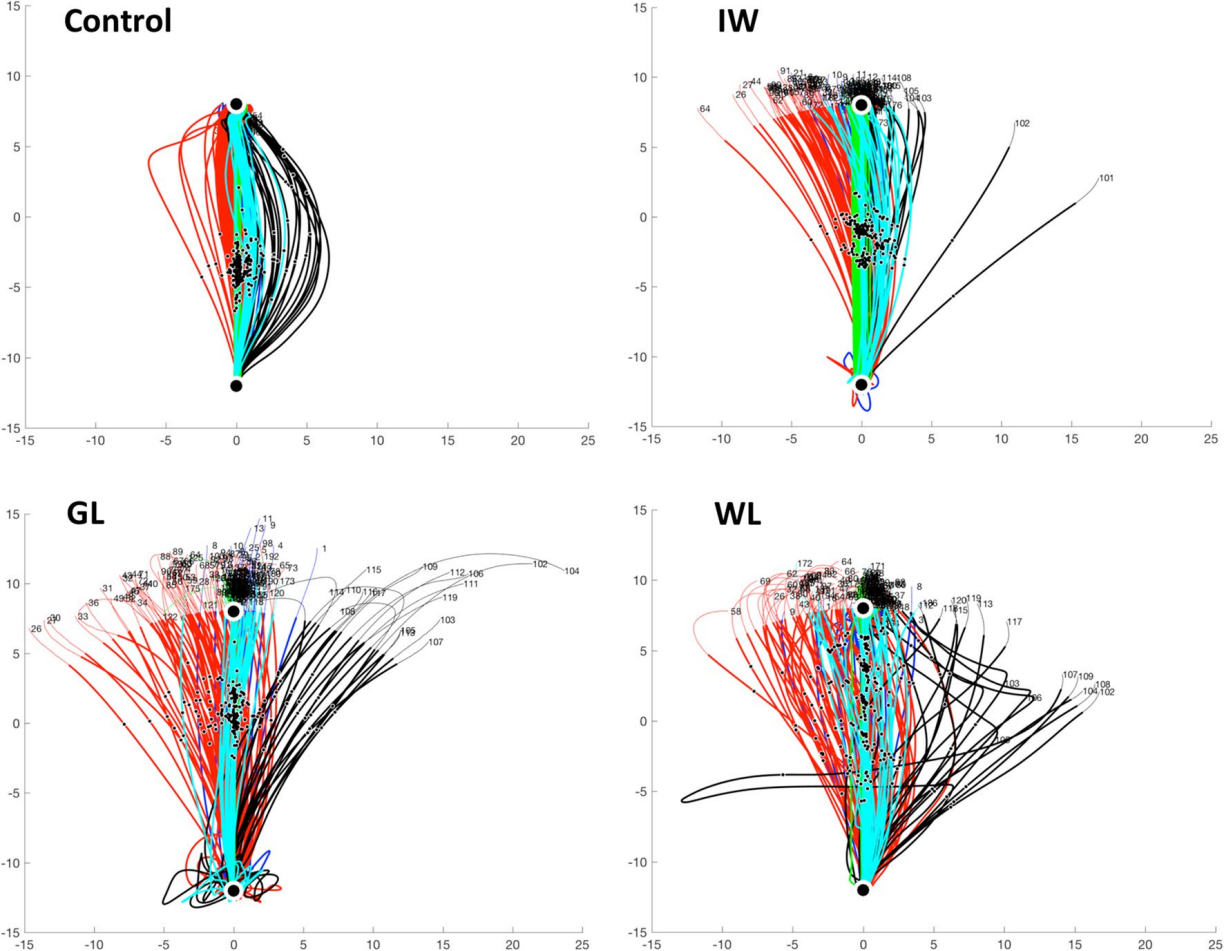
Experiment 2, movement trajectories for a typical control and three PL participants. Each trace is the outward movement from the start location (at bottom of each panel) to the target at the top (large black dot); the axes are in centimetres, relative to the centre of the workspace. For display, the full traces are shown in fine line, but the component used for analysis, shown in thicker lines, terminated when the reach distance exceeded the target distance. The 5 phases of the experiment are colour coded: baseline-blue; primary adaptation-red; secondary adaptation-black; retention-green and washout-cyan. Individual traces show trial number (the time course across trials is shown in Fig. 5). Small black dots indicate the moment of peak velocity. Note that the initial curl-field trials (red) deviate the trajectories anti-clockwise (after left–right normalization for handedness), and for the controls, result in an online corrective action. The reverse curl-field (black) then results in large deviations clockwise. Neither IW nor GL show any online corrective action until late in the movement; the “bounce” shown in some of WL’s movements appeared to be targeted but most movements were not accurately corrected; she dropped the handle on one trial, resulting in its sharp leftward motion

Figure 3a shows that on initial exposure, the hand path for all three participants was noticeably deviated in the direction of the curl field; individual trajectories for IW, GL and WL were like those shown in red in Fig. 4, from Experiment 2. In the single-adaptation experiment, there was clear evidence that all three PL participants could compensate for the curl-field force, with both a reduction in the trajectory deviation during the second half of the curl-field trials (37 trials, Fig. 3a), and with a rise in the compensatory force during channel trials (Fig. 3b). This compensation was maintained during the set of 10 retention trials, to varying degrees between PL participants. The adaptive features then declined in the wash-out phase, with IW and GL returning to baseline performance, although WL showed an after-effect in the wash-out trials, deviating by more than 15° from the target direction, and with high variability across these trials (Fig. 3a). Note there were no control participants in Experiment 1. This experiment demonstrated that all three PL participants could adapt to new dynamics with limited visual feedback of hand position. A more detailed description of the adaptive performance follows for Experiment 2.

### Results: Experiment 2

#### Double adaptation

The double-adaptation protocol has been used to assess the parameters of the slow and fast processes thought to underlie sensorimotor adaptation (Smith et al. 2006). Here, we compared the performance of the PL individuals against a control group of healthy volunteers. As expected from this task, participants initially moved with quite straight-line trajectories towards the target (blue data in Fig. 4). On first exposure to the force field, they were deviated laterally (red traces, Fig. 4). It is striking that the control participants corrected online for this perturbation, typically recovering to reach close to the target even on the first perturbed trial; in contrast, the deafferented participants’ movements showed no evidence of online corrections. The PL participants were clearly (and vocally) surprised by the first exposure trial, despite experiencing similar trials in Experiment 1. Over subsequent trials all participants progressively directed their movements closer to the target. Then on exposure to the reversed force-field, substantial errors in the opposite direction were seen (Fig. 4, black traces), and again there was no strong evidence of purposeful online correction in PL participants IW or GL, in contrast to control participants. The retention phase consisted only of channel trials, with straight-line movements to the target (Fig. 4, green traces); and the final wash-out phase (Fig. 4, cyan) showed behaviour similar to the baseline, with gently curved and reasonably accurate movements.

**Fig. 5:**
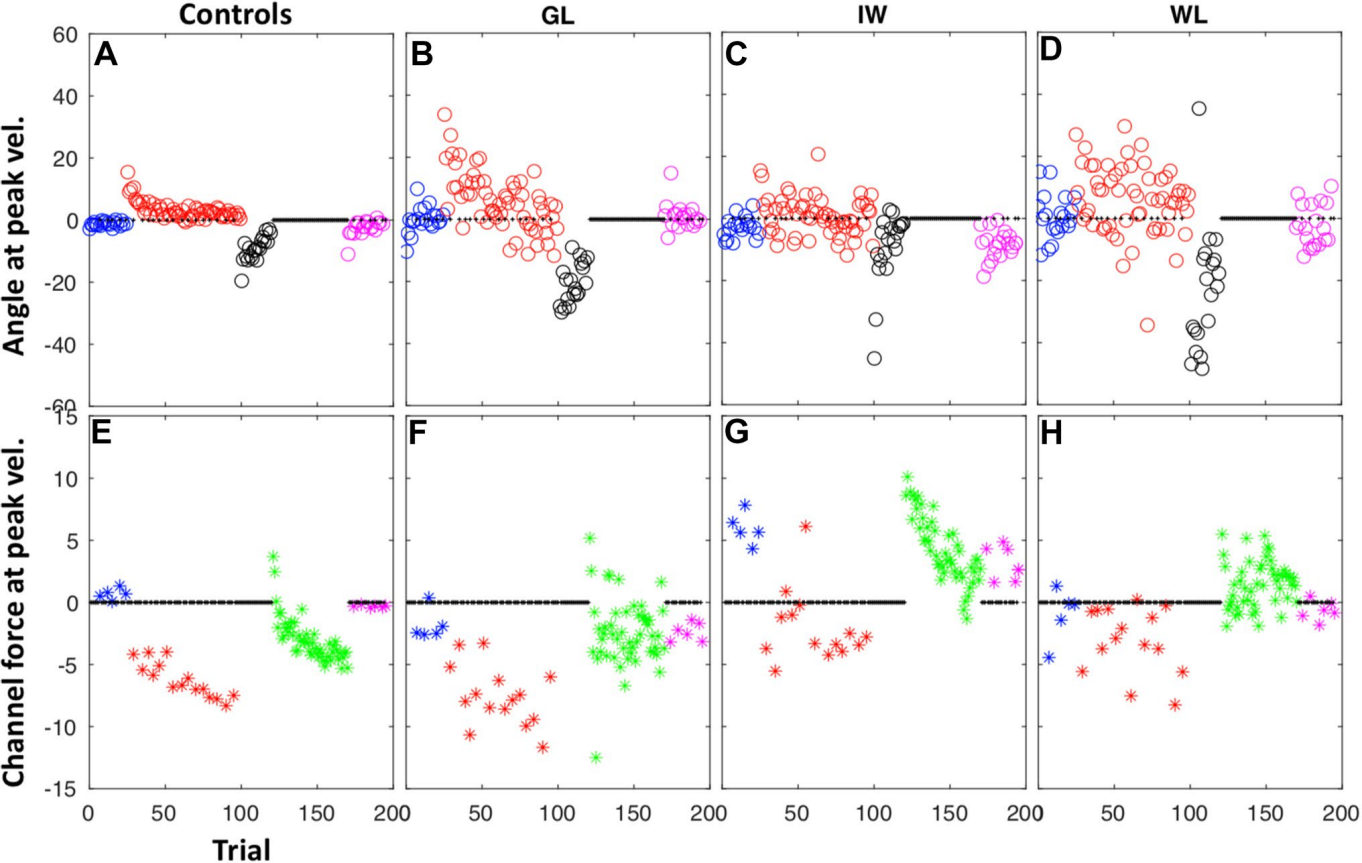
Trial-by-trial responses in double-adaptation, Experiment 2, for the control group (mean, *n* = 7) and three PL participants, GL, IW, and WL. Note that data have been left–right normalised to account for participant handedness. The colour code represents the 5 phases of the experiment, as in Fig. 4. *Upper panels* show the angular deviation at peak velocity, measured in visual feedback trials. *Lower panels* show the normalised force against the channel wall at the moment of peak velocity, measured in channel trials presented 1:5 trials pseudo-randomly (small black crosses) except during the retention phase (green), when there were only channel trials (i.e., no visual feedback trials). There were no channel trials in the reverse-direction secondary adaptation phase (black)

**Fig. 6:**
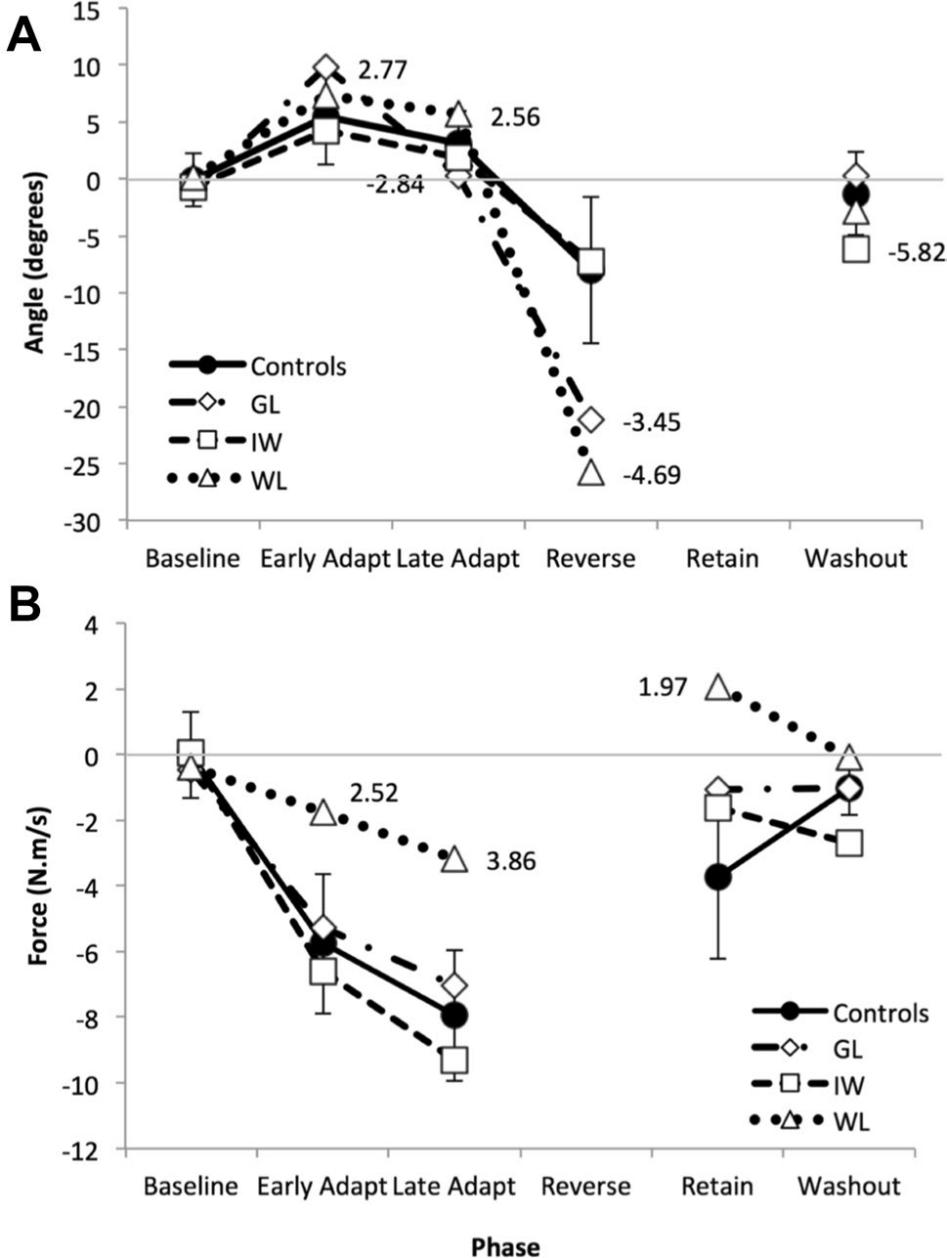
Experiment 2. **a** Mean deviation of the reaching trajectory, measured in visual feedback trials across the double-adaptation experiment. The primary adaption phase was divided into two halves, early and late; there were no visual feedback trials in the retention phase, but only channel trials. Each broken line represents the mean for one PL participant. The solid line is the control group mean (*n* = 7); note that error bars represent ± the mean of individuals’ SD for each phase, to indicate within-phase variance. Numbers indicate *t* scores for individual PL participants relative to the control group mean and control group SD, for those data, where *t* > 1.96. Each individual’s data has been normalised to their baseline, measured as mean performance in the last 12 of the 25 baseline trials. **b** Compensation for the curl-field force, as measured in channel trials presented across the experiment. Format as in Fig. 6a; there were no channel trials in the reverse direction, secondary curl-field adaptation phase

Figure 5 shows the two main performance metrics for each trial, and Fig. 6 shows the mean performance data averaged across trials within each phase of the experiment. The control group showed a reduction in lateral deviation between the first and second half of the adaptation phase (37 trials each, Figs. 5a, 6a), an increase in compensatory force (Figs. 5e, 6b), and deviation in opposite direction during the secondary, reverse-direction curl field, which rapidly decreased to zero (Fig. 5a, black dots). During the retention phase, consisting of 50 contiguous channel trials, the control group showed a gradual increase in force against the channel wall (Fig. 5e, green stars), the “rebound” attributed to the slow memory component (Smith et al. 2006). The PL participants’ overall performance showed similar features but was considerably more variable, with large trial-to-trial variation (Fig. 5b–d, f–h). We computed the standard deviation of the initial movement direction across the baseline phase and the early and late halves of the first adaptation phase. *T* tests confirmed that each PL individual had greater movement variability than the controls in each of these phases (*p* < 0.01 for GL; *p* < 0.05 for IW; *p* < 0.005 for WL).

As in Experiment 1, the PL individuals were quite idiosyncratic in the retention phase (Fig. 5f–h). GL showed no consistent pattern across these trials (Fig. 5f), with a small mean force in the same direction as in the late adaptation phase; WL also showed little or no retention of the forces (Fig. 5h), and despite high variability, her mean force was in the opposite direction to that in the late adaptation stage. IW showed a systematic trend to reduce these forces (Fig. 5g); note, however, that he demonstrated a strong bias in the baseline phase (blue stars), unlike the other participants, so this reduction in force appears similar to the “rebound” effect seen for controls.

**Fig. 7:**
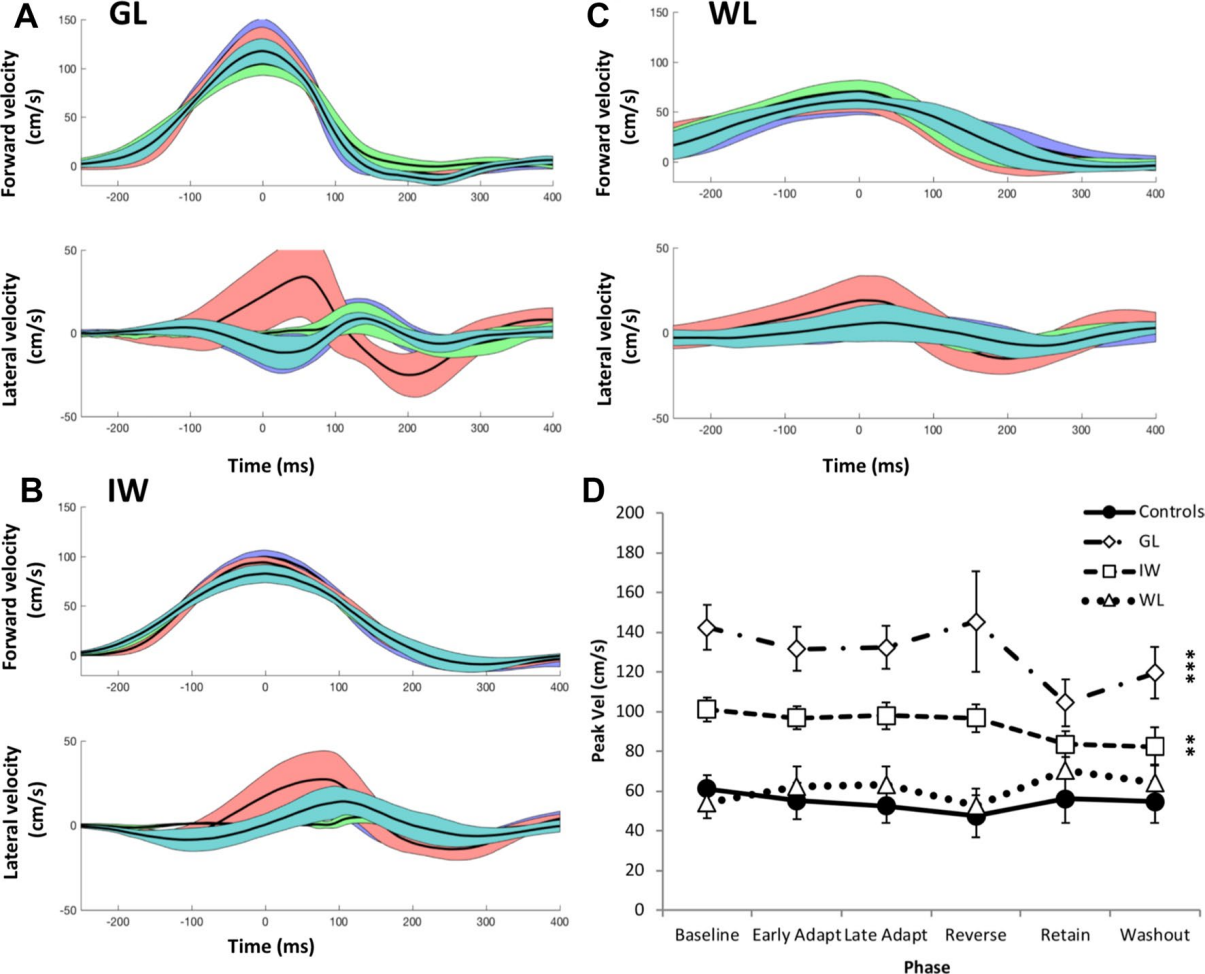
Experiment 2: average velocity of the reaching trajectories. **a**–**c** Forward (upper trace) and lateral velocity (lower trace) profiles for baseline, first adaptation, retention and wash-out phases (black line is mean for each phase ± 1 SD, all traces aligned to peak forward velocity, with colours as in Figs. 4, 5). **d** Mean peak forward velocity across all phases of the experiment. The solid line is the control group mean (*n* = 7); error bars here represent ± the average SD of the within-phase variation across the group. For PL participants, the error bar is the SD of their peak velocities. Asterisks indicate significant *t* test scores for PL participants relative to the control group mean: ***p < 0.005, **p < 0.05, for all phases

Given the velocity-dependent force field, we verified that movement velocity remained constant across the experiment for all participants. Figure 7 reveals that participants were consistent in terms of movement speed and overall velocity profile. The forward velocity had the expected bell-shaped profile for all three PL participants (Fig. 7a–c). WL moved with about the same peak velocity as the controls, on average in all 5 phases (*t* tests, *p* > 0.12). Both GL and IW moved significantly faster than the controls (Fig. 7d, *t* tests in all phases < 0.019); GL noticeably slowed down in the retentions phase, where the visual feedback was presented as an arc representing distance travelled. GL also showed high variability of velocity in reverse adaptation (*p* < 0.0001, which survives Bonferroni correction), whereas IW and WL were more consistent (*p* < 0.03, not significant after Bonferroni correction).

These individual and idiosyncratic variations in performance after proprioceptive loss may be important to consider, in terms of the relationship between trial kinematics and learning (Wu et al. 2014; Seidler et al. 2015; Lefumat et al. 2015), and their individual extent of deafferentation and subsequent recovery of motor function.

#### Modelling

We applied state-space models based on Smith et al. (2006) to the channel trial data from the double-adaptation experiment, to estimate 4 learning parameters, and used model comparison (the difference in Bayesian Information Criterion, BIC; Vrieze 2012) against a single-rate model with 2 parameters (learning and retention rates). As expected (Smith et al. 2006), for all seven control participants there was very strong evidence for the dual-model providing a better fit to the channel trial data (difference in BIC ranged between 28 and 209, average 95.2). The same was true of IW and WL (BIC difference of 32 and 38, respectively). For GL, however, the evidence for the dual-rate model was equivalent to the single-rate model (BIC difference of − 2.7). Table 1 shows the fitted dual-rate parameters. It is noteworthy that the fast retention rate estimated from the channel trials was significantly lower for GL than for the controls (*t* = − 2.07), while the fast learning rate was high for both GL and WL (but not statistically significant; *t* = 0.80 and 1.58, respectively). In addition, WL had a very high slow learning rate, similar to her fast learning rate, which suggests somewhat unstable model fitting at the individual level (with r-squared of only 47%).

**Table 1:** Model parameters for the state-space model fits to channel trial data (top panel), for individual participants including the control group (*n* = 7) and three PL participants

	Group mean	Group SEM	GL	IW	WL
**Fitting on channel trials**					
Fast retention rate	0.75	0.07	**0.40**	0.89	0.61
Fast learning rate	0.20	0.14	0.49	0.04	0.77
Slow retention rate	1.00	0.00	1.00	1.00	1.00
Slow learning rate	0.01	0.00	0.00	0.01	**0.77**
*R*-squared	56%	8%	46%	57%	47%
**Fitting on feedback trials**					
Fast retention rate	0.48	0.13	0.29	0.93	0.74
Fast learning rate	0.30	0.06	0.28	0.38	0.31
Slow retention rate	0.98	0.01	1.00	1.00	1.00
Slow learning rate	0.09	0.05	0.05	0.03	0.06
Scaling function/100	0.12	0.01	**0.21**	**0.22**	**0.33**
*R*-squared	64%	4%	76%	56%	49%

In the lower panel the model is fit to the feedback trials only, requiring an additional scaling factor. Values highlighted in bold indicate that PL participants’ parameters have a *t* score of > 1.96 with respect to the control group

To further estimate adaptation rates, we separately fitted the deviation data during feedback trials with similar dual-rate and single-rate models; an additional free parameter accounted for the idiosyncratic scaling of the curl-field forces to the magnitude of the deviation, which could be influenced by limb stiffness, arm mass and other factors. In this case the BIC evidence in favour of the dual-rate model was very strong for all three PL participants (BIC difference > 29), and for 6 out of 7 controls (BIC differences of 7.5–68.3; for the exception, the evidence for the models was about equal with a BIC difference of − 0.01). There was a trend for IW and WL to have a higher fast retention rate than others, while GL had a lower fast retention rate, but these differences did not reach statistical significance (*t* < 1.96). The additional scaling parameter was significantly higher for all three PL participants compared to the controls, reflecting that the lateral deviation of the PL participants’ movements during the curl-field trials was greater (see also Fig. 5, top). WL had a somewhat higher learning rate for the slow component, and again a rather low model fit, with R-squared of 49%.

#### Predicting individual performance

Because of the known relationship between baseline variability of movement kinematics (Wu et al. 2014) and adaptation, and the relationship between both movement speed and positional variability and inter-manual transfer of adaptation (Lefumat et al. 2015), we tested the relationships between these variables and metrics of adaptation, using the difference between early and late primary adaptation, reverse adaptation, and retention. The controls showed a significant positive correlation between baseline mean speed and the peak forces generated in the retention phase (the “spontaneous recovery effect”, Smith et al. 2006; *r* = 0.81, *p* = 0.027). This was not correlated across PL participants (*n* = 3, *r* = 0.66, *p* = 0.54), and the regression was insignificant when tested across all participants combined (controls and PL; *n* = 10; *r* = 0.05, *p* = 0.81). In contrast, for the PL participants, the amount of adaptation in the primary phase (late performance minus early performance) was positively predicted by their baseline mean speed (*r* = 0.994, *p* = 0.070) and negatively predicted by variability of baseline movement direction (*r* = − 0.99, *p* = 0.072). Note in both cases the low sample size, *n* = 3, defeating statistical significance. The positive relationship between baseline speed and adaptation was significant for the combined group (*n* = 10; *r* = 0.82, *p* = 0.004). The negative relationship between baseline variability and adaptation was not significant for the control group alone (*n* = 7; *r* = − 0.53, *p* = 0.22), or for the combined group (*n* = 10; *r* = − 0.30, *p* = 0.39).

In summary, we saw significant adaption to curl-field forces in three deafferented participants, but with idiosyncratic patterns of behaviour in channel trials. Modelling their performance suggests their learning rates are generally not significantly different from the range seen in a control population, but they show unusual behaviour in the retention phase, and they show unusually large lateral deviations in the face of the curl-field perturbations, generally without online correction.

## Experiment 3: detection of force fields

We next sought to measure the ability of the PL participants to detect the force fields, both with a cursor showing the hand trajectory (as in Experiments 1 and 2) and in the absence of cursor feedback. We predicted normal performance in the condition with a visual cursor. In the condition without a cursor, we hypothesized that PL participants should have very low performance as they are deprived of both visual and proprioceptive feedback from the moving arm.

### Methods

#### Deafferented participants

All three participated, in testing sessions held during their 2-day visits for the force-adaptation experiments.

#### Control participants

Eighteen age-matched controls also participated in the experiment, with 3 groups of 6 each age-matched to one PL participant; none of these controls had participated in Experiment 2. One control’s performance was an extreme outlier on all measures and was excluded from further analysis. The remaining control groups were matched to IW (*n* = 6, 61.7 ± 2.9 years, 4 males, 6 right handers), GL (*n* = 6, 67.2 ± 2.9 years, 3 males, 4 right handers) and to WL (*n* = 5, 48.8 ± 4.0 years, 4 males, 5 right handers; Oldfield 1971); one participant had a laterality quotient of zero, and was classified by his stated hand preference.

#### Behavioural task

During each trial, participants were subjected to a clockwise (CW) or counter-clockwise (CCW) curl field, which they were instructed to try to counteract to reach the target. When the participant had moved 15 cm in the sagittal plane, the target turned green and the trial was completed once hand velocity fell below 3 cm/s. The participant then had to verbally indicate the perceived direction of the perturbation. Once this two-alternative forced choice (2AFC) response was recorded, the start target reappeared, the handle was guided back and the next trial began. If movement velocity fell below 2 cm/s before the reach extended 15 cm in sagittal plane, or if the movement took less than 300 ms or more than 1200 ms, the trial was aborted, with feedback provided on-screen to inform participants whether they were too fast or slow. Trials were performed with or without visual feedback, on a pseudorandom basis, with the strength and direction of the curl-field controlled by 4 interleaved psychometric sequences (Taylor and Creelman 1967).

#### Psychometric sequences

For both vision and no-vision conditions, one staircase started with leftward (CCW) forces, the other with rightward (CW) forces. Field strength increased if the last response was incorrect and reduced if it was correct. Each sequence began at 15 N.m/s, and was capped at a maximal limit of 18 N.m/s. A reversal was defined as consecutive correct and incorrect responses (or vice versa).

Three variations of staircase sequence were used to optimise data collection for the individual PL participants, summarised below; we, therefore, recruited separate control groups for each PL participant, who performed matching tasks.

#### Participant GL and 6 controls

Each change in force level was either a 40% increase or decrease from the previous field strength. A correct response had to be provided twice before progressing to the reduced field strength, whilst only a single incorrect response was needed to increase the force level. The experiment terminated after each staircase had reversed direction 15 times.

#### Participant WL and 5 controls

WL fatigued quite quickly. Hence only a single correct or incorrect response was taken to increase or decrease the field strength by 40%, respectively, and the experiment terminated after 180 trials were completed (maximum possible 15 reversals).

#### Participant IW and 6 controls

To ensure rapid approach towards the threshold and then finer testing near threshold, field strength FS varied on every trial. While FS > 2 N.m/s, each change was a 40% increase or decrease of the previous level. For FS < 2 N.m/s, the following equation was used:

2 $${\text{F}}{{\text{S}}_{{\text{New}}}}=\alpha \times {\text{F}}{{\text{S}}_{{\text{Current}}}}+\left( {\alpha - 1} \right) \times {\text{F}}{{\text{S}}_{{\text{Initial}}}}$$

For correct responses, *α* = 0.97; for incorrect responses *α* = 1.02. The experiment terminated after each staircase had reversed direction 15 times or after 180 trials.

#### Post-hoc analysis

The verbal responses were coded (“right” = 1, “left” = 0) and plotted against the lateral force experienced at peak velocity during the reach (i.e., *f*_*x*_ = FS × *v*_*y*_ see Eq. 1). The vision and no-vision conditions were analysed separately. A logistic function was then fitted to each participant’s data, combining responses from the two staircase sequences, using the Matlab *glmfit* function. Any data points with Pearson’s residuals greater than 2SD from the curve were eliminated and the curve refitted. The bias was then defined as the 50% probability point of the logistic function; the uncertainty range was determined by the inter-quartile range (25–75%). Bias represents a systematic error in the perceived force direction; uncertainty range is equivalent to the just-noticeable difference limit: the magnitude of forces that are detectably different. Because of non-normal control group data, Mann–Whitney *U* tests were used to determine the significance of the differences in uncertainty and bias between the deafferented and control groups. To examine any potential differences arising from the three psychometric protocols, bias and uncertainty were compared between the 3 control groups using a one-way Kruskal–Wallis test, performed separately for the vision and no-vision conditions.

### Results

We first examined the control group results. There were no significant differences between the three control groups (vision: bias *χ*^2^[2] = 2.63, *p* = 0.280; uncertainty range: *χ*^2^[2] = 0.45, *p* = 0.813; no-vision: bias *χ*^2^[2] = 1.93, *p* = 0.403; uncertainty range *χ*^2^[2] = 5.00, *p* = 0.078), suggesting that our pragmatic changes to the staircase procedures to accommodate the PL participants did not influence our measurements. Bias was greater with vision (Wilcoxon signed-rank test, *n* = 17, *p* = 0.008), but uncertainty did not differ with and without vision.

**Fig. 8:**
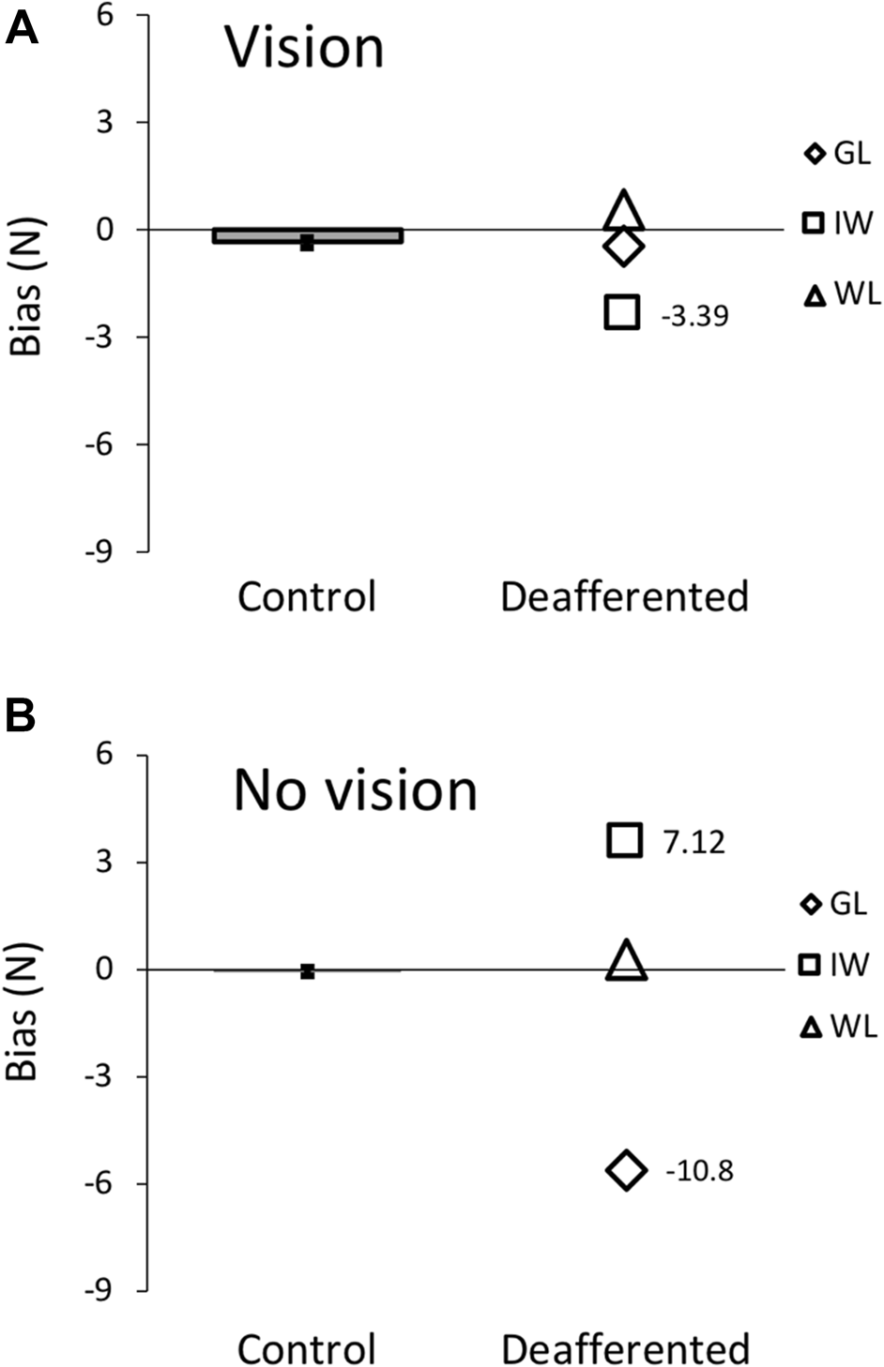
Experiment 3: force-detection thresholds. Bar (mean ± 1 SD) and scatter plots for the combined control group (*n* = 17 participants) and three PL participants, respectively, for bias in the vision (**a**) and no-vision condition (**b**). The numbers indicate, where the *t* score for PL participants’ bias was > 1.96 with respect to the control group. GL and IW individually had bias values significantly different from the controls, but in opposite directions

**Fig. 9:**
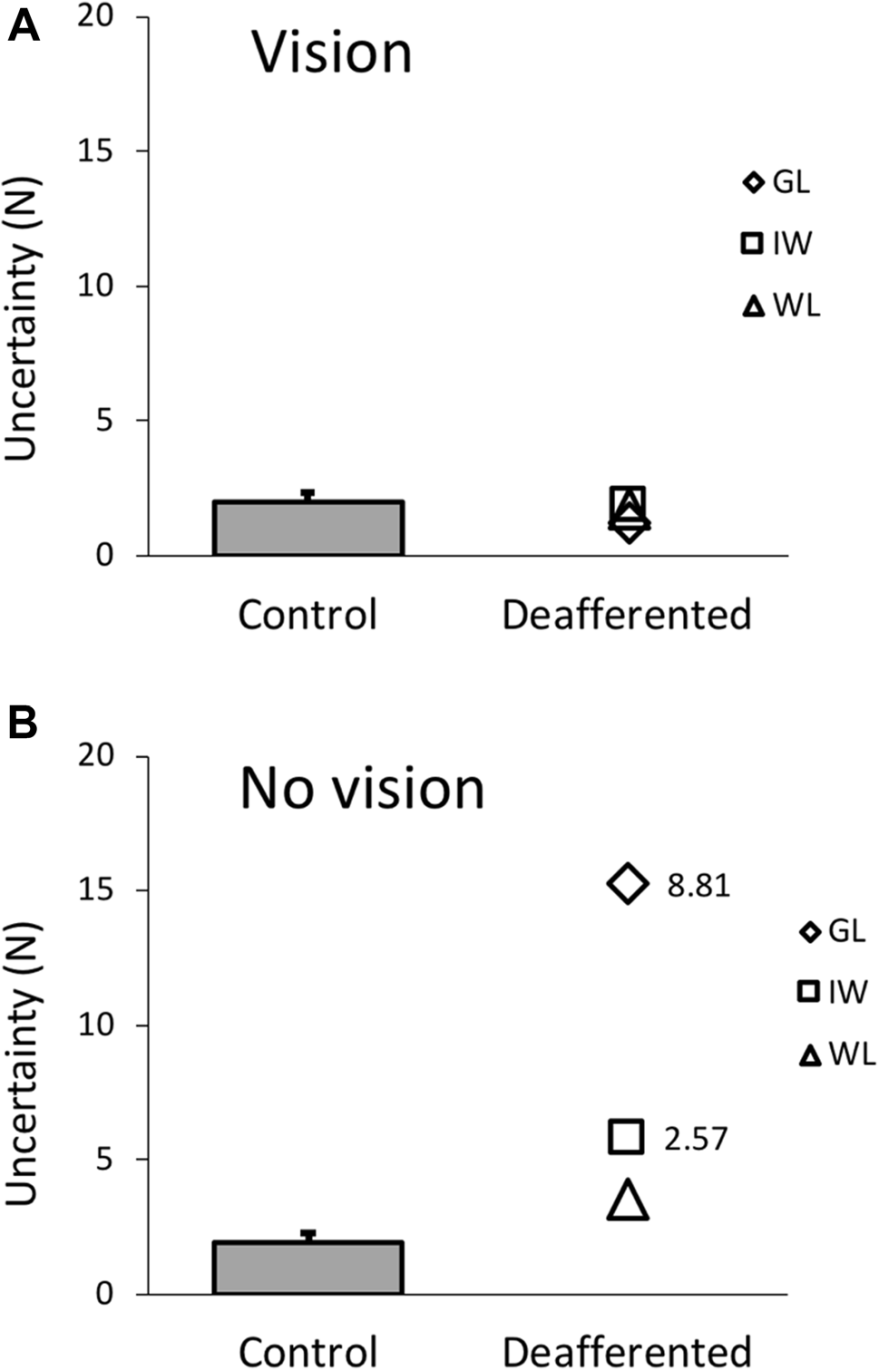
Experiment 3: bar (mean + 1 SD) and scatter plots for the uncertainty range for the set of three combined control groups (*n* = 17) and three PL participants, respectively, in the vision (**a**) and no-vision condition (**b**). The numbers indicate, where the *t* score for PL participants’ uncertainty range was > 1.96 with respect to the control group

The perceptual data comparing the PL and control group are summarised in Figs. 8 and 9. At the group level, Mann–Whitney *U* tests showed there were no significant group differences for bias in either vision (*U* = 24.0, *p* = 0.921, not shown) or no-vision conditions (*U* = 21.0, *p* = 0.689; Fig. 8). Because we have no prior reason to expect a shift in either direction, but the bias might be related to handedness, we also compared the absolute bias values: absolute bias did not significantly differ between PL and control groups for either the vision (*U* = 14.0, *p* = 0.258) and no-vision (*U* = 9.0, *p* = 0.093) conditions. The uncertainty range was also not significantly different between groups in the vision condition (*U* = 23.0, *p* = 0.842), but there was a significant difference between groups in the no-vision condition (*U* = 3.0, *p* = 0.012, Fig. 9). The PL group had larger uncertainty ranges than controls (median of 5.81N vs 1.26N, respectively, and means of 8.21 N ± 6.20 (SD) vs 1.92 N  ± 1.51). However, the heterogeneity of the three PL participants suggests that group statistics are inappropriate.

To compare the PL participants individually against controls, standardised *t* scores were calculated using the mean and standard deviation from the combined control population. In the vision condition, all three PL participants were similar to controls for uncertainty range in the vision condition, and WL and GL were also similar to the controls in their bias. However, IW had a significantly larger, more negative bias (*t* = − 3.39, *p* < 0.001).

Unexpectedly, in the no-vision condition, WL’s performance was comparable to controls for both bias and uncertainty range, whilst both GL and IW had significantly larger uncertainty ranges (GL *t* = 8.81, *p* < 0.001; IW *t* = 2.57, *p* = 0.010) and larger biases (GL *t* = − 10.80, *p* < 0.001; IW *t* = 7.12, *p* < 0.001) than controls. The bias shown by IW may be related to the bias he showed in channel trials on Experiment 2 (Fig. 5g).

**Fig. 10:**
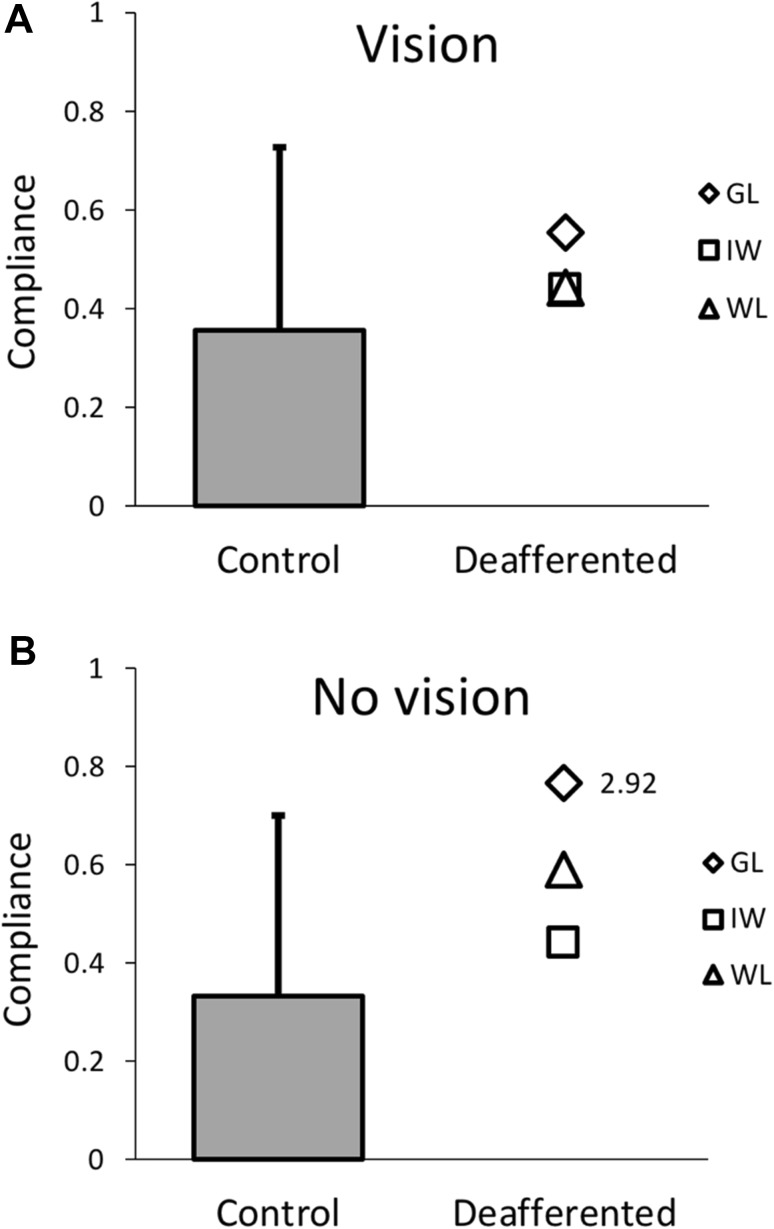
Experiment 3: bar (mean + 1 SD) and scatter plots for the estimates of limb compliance for the control group (*n* = 17) and the three PL participants, respectively, in the vision (**a**) and no-vision condition (**b**). GL’s compliance was significantly greater in the no-vision condition (*t* = 2.92). See main text for compliance estimation

To estimate differences in limb stiffness between PL and control groups, the lateral force experienced at peak velocity was regressed against the magnitude of concurrent lateral deviation from a straight path linking start position to target. The resultant gradient of this regression model was then taken as an index of limb compliance (inverse of stiffness), since a steeper gradient indicates larger deviations and hence a more compliant limb. On an individual level, the PL participants had somewhat higher than average compliance in both conditions (Fig. 10); however, *t* scores showed that only GL had a significantly more compliant limb in the no-vision condition (*t* = 2.92, *p* = 0.004). These results suggest that limb stiffness was not likely to influence the PL group’s detection of force perturbations at least in the vision condition.

## Experiment 4: head motion

Intrigued by the force-field detection of the deafferented participants, we sought to test whether the standard curl-field forces applied in Experiment 1 and 2, and the quite small forces applied in the psychometric testing of Experiment 3, resulted in motion of the upper limb, neck or head that might provide non-visual sensory cues for participants.

### Methods

#### Deafferented participants

None of the PL patients were available for this experiment.

#### Control participants

Six healthy adults (34.5 ± 12 years, 4 males) participated in the experiment; all were self-declared right handers. None had participated in the previous experiments.

#### Behavioural task

We implemented the same force-detection task as described above in Experiment 3, using the sequence for IW. However, only 100 trials were completed, as final psychophysical thresholds were not required. The first 20 trials were practice, and the participants familiarised themselves with the behavioural task and the required 2AFC responses (see Experiment 3). Then three motion tracking markers (Polhemus Liberty, 240 Hz sample rate) were attached to the back of the hand, to the forearm near the elbow, and to the shoulder. An additional marker was attached to a cloth baseball cap, such that the marker was centred at the back of the head. The fifth marker was attached directly to the vBOT handle, to allow co-registration of the individual trials recorded by the vBOT control program and the continuous record of the motion tracking data from the participant. Eighty trials of the perceptual task were then completed, 40 with and 40 without vision pseudo-randomly interleaved, as in the previous Experiment 3.

#### Analysis

We collapsed data across both vision and no-vision trials. To test the relationship between hand and head movement and the applied force perturbation, the lateral hand position, and the lateral and forward head velocity were detected at the moment of peak hand velocity (when the force perturbation was maximal). The median values of hand position and head velocity (either lateral or forward velocity, analysed separately) were found for each participant. Each trial was then coded as 1 or 0, if the hand position and head velocity was above or below the median, respectively, and these codes were used as a proxy “perceptual” response. Logistic functions were then fitted to responses across the range of applied peak forces, in the same way that the verbal responses in Experiment 3 were analysed. The uncertainty range was taken as a measure of the fidelity with which that participant’s hand or head motion could be used to determine the applied force direction.

### Results: Experiment 4

For all six participants, there was a reliable relationship between hand position at the moment of peak force and the applied force direction, as determined by the width of the psychometric function: the average uncertainty range (the inter-quartile of the logistic curve) was 1.22 N (± 0.72, SEM), similar to the range seen for the controls in the no-vision condition of Experiment 3 (Fig. 9). Thus, it would be possible for an ideal observer to determine the direction of the applied curl–force from only the deviation of the hand; control participants might be expected to gain this information from proprioception. Four of the six participants also showed a reliable correlation between the direction of head velocity and applied force direction, with an uncertainty range for these four of 4.88 ± 0.73 N for lateral head velocity, and 5.5 ±1.2 N for forward head velocity. These are of the same magnitude as the uncertainty range seen in two of the PL participants tested in Experiment 3 (IW and WL, Fig. 9). This also suggests that an ideal observer who has no access to information about the direction of hand deviation could, in 4 of 6 cases, determine the curl-field direction from head motion, detected either through cervical afferents or vestibular signals.

## Discussion

We have demonstrated that three participants with chronic proprioceptive loss (PL) were able to adapt their seen reaching movements to velocity-dependent curl fields (Experiment 1), showing a reduction in the lateral deviation caused by the perturbing force. They actively compensated for the expected perturbation, pushing against the virtual walls in channel trials. We also found that they showed similar accuracy to controls in detecting the perturbed forces in trials with visual feedback, and could even detect forces (albeit at a higher level) without visual feedback. We speculate that this might be due to the reactive forces on the body, leading to small but reliably detectable head motion. By comparing their adaptation to two successive opposite direction curl fields with a control group (Experiment 2), and using state-space modelling of their responses, we were able to assess their learning and retention rates. As hypothesized, while the modelled learning parameters were generally within the normal range, the PL participants GL and WL had fast learning rates somewhat higher than the controls; GL also had a lower fast retention level compared to the controls. This implies that GL and WL were more reactive to trial-to-trial errors and showed relatively poor retention of the adaptation. In contrast, against our hypothesis, participant IW trended towards a lower learning rate than controls (estimated from the channel trial data) and towards higher than normal retention.

### Adaptation to forces

While the ability of deafferented individuals to adapt to new limb dynamics with full or limited visual feedback has already been shown (Sarlegna et al. 2010; Yousif et al. 2015; Lefumat et al. 2016), our data suggest that they may use different adaptive mechanisms compared to controls, with faster learning rates and weaker retention (GL and WL) suggesting a strategic change (Taylor et al. 2014). This would fit with their greater dependence on cognitive control of movement in the absence of proprioceptive feedback (Ingram et al. 2000), and in turn this implies that they are well aware of the perturbations, perhaps consciously detecting trajectory errors caused by the curl forces based on non-visual and/or visual cues.

### Detection of forces

We assessed participants' ability to consciously detect the perturbing force field and Experiment 3 showed that all three PL participants were able to detect the direction of the perturbing forces trial-by-trial, with or without a visual cursor, at force thresholds well below the 10 N.m/s level used in the first two experiments. The latter perceptual performance (detection without proprioception or visual feedback from the moving limb) is remarkable. Unlike the data reported by Yousif et al. (2015), who tested IW’s static and active position sense, in our task the PL participants were as good as the controls when vision of the cursor was allowed (Figs. 8a, 9a), but worse without (Figs. 8b, 9b). Yousif et al. (2015) reported that IW had apparently a better active proprioceptive acuity (inter-quartile range, IQR = 16°) than controls (IQR > 45°) although this very high uncertainty for their healthy control group is much higher than other reports (Cressman and Henriques 2009; Ostry et al. 2010; Wong et al. 2011).

The minimum change necessary to accurately detect the direction of the stimulus (the just-noticeable difference or “JND”) is represented by half of the inter-quartile range of a psychometric curve, which we term the uncertainty range. Hence, with an average uncertainty range in Experiment 3 of 8.21 N (± 3.6 N SEM) in the no-vision condition, the PL participants were able to reliably detect force changes of about JND = 4.1N. While worse than the controls (JND = 0.96 N ± 0.76, SD), this is remarkable performance in the absence of any known somatosensory feedback from their unseen, moving limb. It is possible that they could detect low forces in the perceptual task (Experiment 3) because of the higher consistency of their movements, as we constrained the movement times more tightly than we did in the adaptation task (Experiments 1 and 2), and so the velocity-dependent perturbations were also more constrained. However, the thresholds reached were well below the curl-field constants used in the adaptation tasks (10 N.m/s), implying that PL participants could efficiently detect the onset and the direction of the curl fields.

### Limb stiffness

One potential strategy by which PL participants may have been able to detect force perturbations in the no-vision trials is by increasing stiffness of the unseen limb, and then detecting reaction forces with intact afferents from the neck or from the vestibular system. The PL participants all have intact vestibular function and IW has some residual neck sensation, but GL and WL do not (WL does have some temperature sensation from the neck). It has been shown that neurologically normal participants deviate their reaching actions if the vestibular system is stimulated, or the head rotated on the trunk (Guillaud et al. 2011; Blouin et al. 2015). However, our estimates of limb stiffness in the PL participants suggest they were not unusually stiff, an observation consistent with the large lateral deviations seen in Experiments 1 and 2. In fact, on the first few curl-field trials, they were deviated considerably more than controls (up to 30°–45°) and all expressed surprise. Likewise, they were surprised by the very large deviations on the first of the reversed-direction curl field, during the double-adaptation experiment (Experiment 2).

### Visual and somatic cues of force direction

Experiment 4 showed that the deviated hand path could reliably provide an ideal observer information about the direction of the curl-field. The similarity between the uncertainty ranges estimated from the hand path in Experiment 4 and in the visual feedback condition for the PL participants (Experiment 3, Fig. 9) suggests that they might have been able to use visual feedback to detect force direction. This is not trivial, however, as proprioceptive loss leads to increased variability of movements (Gordon et al. 1995; Sarlegna et al. 2010). Hence detection of a small visual perturbation requires the ability to contrast the motion of the cursor with the expected movement of the unseen hand. Healthy participants appear to do so, leading to active unlearning in channel trials (Lago-Rodrigues and Miall 2016); PL participants also appear to have sufficient knowledge of their expected movement to make this judgement, despite high trial-to-trial variability. This may lead to their ability to selectively adapt to position- and velocity-dependent forces (Yousif et al. 2015).

Experiment 4 also indicated that in 4 out of 6 normal participants, subtle head motion during the reaching action was correlated with the direction of the curl-field direction, albeit with less acuity than from hand motion. Hence the similarity between the uncertainty range estimated from lateral head motion in the controls and the decisions for the PL group in the no-vision condition (Fig. 9) suggests that they might have been able to use cues from the head and/or neck to detect the force direction. This might include cervical proprioception (for IW only; Cole and Paillard 1995), vestibular input (Blouin et al. 2015), or visual detection of whole scene motion (Saijo et al. 2005).

In the channel trials, PL participants had feedback of movement extent from the arc cursor, and they had no visual feedback of the constrained movement path. They may still have been able to assess the reaction of their compensatory forces against the channel walls by vestibular or neck afferents. Unfortunately, we did not have the opportunity to record head motion in the three deafferented participants; this is something that should be done when possible.

### Strategic retention

Knowledge of the perturbations would allow a strategic shift in movement control and may explain the quite high learning rates and low retention rates shown by GL and WL. In essence, they respond strongly to recent errors, and retain relatively little of the learning. Hence in the channel trials of the retention phase, when normal controls demonstrated a spontaneous recovery of the previous adapted state (Fig. 5e, and Smith et al. 2006), at least GL and WL did not. IW, on the other hand, showed learning rates closer to the controls, and was more consistent in his responses. He also appeared to show a gradual shift in the retention phase (Fig. 5g, green), that might be the sign of a memory rebound, given the high bias in his baseline (Fig. 5g, blue), but this interpretation is uncertain. It is interesting to note that IW was the only PL participant to show a pronounced bias in the perceptual task (Fig. 8), and this bias reversed from vision to no-vision trials.

Lackner and DiZio (1994) and Batcho et al. (2016) have shown that adaptation to force fields is possible in proprioceptively intact subjects without any visual feedback (see also Scheidt et al. 2005; Franklin et al. 2007; Lefumat et al. 2015). Batcho et al. (2016) reported that they tend to adapt slower and overcompensate, compared to adaptation with visual feedback, achieving s-shaped trajectories that initially deviate into the force field, i.e., showing greater anticipation of the forces. Izawa et al. (2008) reported this as a dynamically optimal strategy. There may well be different strategies at play, in the presence and absence of visual feedback. Online visual feedback promotes more implicit adaptation and is thought to achieve stronger update of internal models (Taylor and Ivry 2011; Taylor et al. 2014; Huberdeau et al. 2015). In the absence of proprioception, however, control is highly dependent on explicit, cognitive processes, and the lack of rebound recovery observed in Experiment 2 for GL and WL is consistent with this idea.

### Online corrections

It was a surprise to us that the stiffness of PL participants (estimated by the lateral deviation of the arm during curl-field trials) is lower than that of the controls. Note, however, that they have no stretch reflexes, and no online correction of trajectories was observed for GL and IW (Fig. 4). Yousif et al. (2015) showed that IW adopted appropriately timed force profiles, even when compensating for forces without directional feedback, although his actions had large “hooks” in the end stage of the trajectory that often brought his arm to a common final location. Few if any of his trials here had obvious hooks (Fig. 4), and IW made almost straight trajectories even on the first few trials in the curl fields. Very large late hooks similar to those reported by Yousif et al. (2015) were seen in some trials for GL and WL (Fig. 4); Sarlegna et al. (2010) have seen the same behaviour in GL in response to Coriolis forces and in baseline conditions. Yousif et al. (2015) suggested that IW’s temporal specific responses (to position or velocity-dependent fields) argue against a strategy of high stiffness and end-point control (Polit and Bizzi 1979). However, we would suggest that together, these data imply that the PL participants may have strategies based on a low stiffness, ballistic “throw” of the arm, coupled with a commanded final posture. We recently reported that IW appears to mix amplitude and position control, for controlling single joint wrist movements in the absence of visual feedback (Miall et al. 2017).

### Kinematics

Finally, our kinematics analysis showed that for the PL participants, the amount of adaptation had a negative relationship with baseline directional variability [the opposite to that expected from Wu et al. (2014)] but a positive relationship with mean speed. There was no significant relationship between baseline kinematics and retention. For controls, there was a positive linear relationship between baseline speed and retention. That baseline variability can predict adaptation is thought to reflect individuals’ propensity to explore the current motor context (Wu et al. 2014). In the face of a perturbation, the prior knowledge of the baseline task gained by exploration, or the willingness to explore during the adaptation phase, could supplement error-driven learning. For the PL participants, however, much of the variability likely reflects weaker control, rather than active exploration, and so the negative relationship may be driven by this factor. Bock and Thomas (2011) also reported reduced adaptation to velocity-dependent forces, and increased variability, when proprioception in normal subjects was disrupted by muscle vibration (see also Pipereit et al. 2006). In the present study, WL had high variability (*t* = 2.7) and both IW and GL were above the control mean (*t* = 1.50 and 0.45, respectively).

It is not obvious why mean velocity should predict subsequent adaptation, but since the perturbations are velocity-dependent, higher mean speeds will lead to larger perturbations, which are potentially easier to detect. GL and IW tended to reach much faster than controls (*t* > 2.3 in all phases of Experiment 2, Fig. 7d), whereas WL moved at normal speed (*t* < 1.25). Despite her lower average speed, WL found the movement tasks difficult, and is less experienced in this type of experiment; in contrast, GL seemed happy to execute large numbers of trials, despite the quite strong perturbations she experienced. We thus hypothesize that the high speeds produced by GL and IW magnified the effect of the curl fields, and allowed substantial adaptation, whereas WL’s low speed and high variability reduced her adaptation potential.

### Individual differences

We end with a reminder that profound proprioceptive loss can either be congenital, progressive, as in some autoimmune conditions, or catastrophically acute and complete, as here with sensory neuronopathy. Research with participants without proprioception is, therefore, with those who have lived with the condition for years—even decades—and have found ways to reduce its effects. They have learnt to compensate and to maximise their use of remaining cues, such as vision and potentially subtle vestibular inputs, but with (at least for IW) a high cognitive cost. Though the three deafferented participants have similar severe sensory loss, their behaviour during these tests, and in daily living, suggests they are not a uniform or homogenous group. It is also important to realise that the everyday motor abilities of these three differ greatly too (see Methods), and this might be of relevance to the interpretation of our experiments.
